# Panacea: Visual exploration system for analyzing trends in annual recruitment using time-varying graphs

**DOI:** 10.1371/journal.pone.0247587

**Published:** 2021-03-01

**Authors:** Toshiyuki T. Yokoyama, Masashi Okada, Tadahiro Taniguchi

**Affiliations:** 1 The University of Tokyo, Chiba, Japan; 2 Panasonic Corporation, Osaka, Japan; 3 Ritsumeikan University, Kusatsu, Shiga, Japan; University of Management and Technology, Pakistan, PAKISTAN

## Abstract

Annual recruitment data of new graduates are manually analyzed by human resources (HR) specialists in industries, which signifies the need to evaluate the recruitment strategy of HR specialists. Different job seekers send applications to companies every year. The relationships between applicants’ attributes (e.g., English skill or academic credentials) can be used to analyze the changes in recruitment trends across multiple years. However, most attributes are unnormalized and thus require thorough preprocessing. Such unnormalized data hinder effective comparison of the relationship between applicants in the early stage of data analysis. Thus, a visual exploration system is highly needed to gain insight from the overview of the relationship among applicant qualifications across multiple years. In this study, we propose the Polarizing Attributes for Network Analysis of Correlation on Entities Association (Panacea) visualization system. The proposed system integrates a time-varying graph model and dynamic graph visualization for heterogeneous tabular data. Using this system, HR specialists can interactively inspect the relationships between two attributes of prospective employees across multiple years. Further, we demonstrate the usability of Panacea with representative examples for finding hidden trends in real-world datasets, and we discuss feedback from HR specialists obtained throughout Panacea’s development. The proposed Panacea system enables HR specialists to visually explore the annual recruitment of new graduates.

## Introduction

Recruitment of new employees is one of the most vital duties in Human Resources (HR) management. HR specialists themselves wish to discover the comparative and chronological trends of applicants from the pool of applicants’ historical data. For example, they wish to compare distributions in the English skills of prospective employees. However, the heterogeneity of the large database requires a great deal of pre-processing before the trend analysis, resulting in actual data loss. A method for gaining insight into the relationships over multiple years of database records is required. An interactive visualization platform is therefore essential for interactive exploration of data by HR specialists.

Data analysis conducted by a company facilitates the evaluation of previous business strategies and the discovery of hidden trends or biases [1]. The importance of data analysis is also recognized in HR management [2, 3]. Among various HR functions, recruitment is one of the most important tasks for growing a company and assigning appropriate personnel to each section, department, etc. For most local companies in Japan, the standard recruitment source is a short annual recruitment period for new graduates each year [4, 5] Most Japanese local companies recruit new graduates during the same short period every year, which is usually a few months. At the same time, students in their final year of school apply to one or more companies during this period. Companies select prospective employees among the applicants through a screening process including a series of interviews. As a result, large companies have to process many applications during a short period. It has been estimated that more than half of university graduates have worked in the same company for more than a decade as a result of the lifetime employment system in large Japanese companies [6]. Therefore, an effective review of prospective employees is a critical task for HR specialists.

Analyzing the large volume of applications and wide variety of applicant qualifications is challenging. Applicants’ data are stored in a recruitment database as records in a table. Different data types are used to store the attributes of applicants’ data in each column of the table in the database (e.g., name as a string, English exam score as a number, or academic credentials as a category, etc.). Attributes fields are sometimes left empty and are not normalized across the table, and attributes have different data types.

We denote this characteristic of unnormalized attributes as *heterogeneity*, which makes it difficult to compare the attributes of applicants across different years. Usually, larger companies receive more than a thousand applications each year, which increases the pre-processing effort. A large number of applicant records are managed in a recruitment management system, but rich analytical functions are excluded from the system’s design. Therefore, HR specialists must manually review the applicants’ data.

Resolving the heterogeneity of attributes involves a lot of pre-processing effort, which is still a challenging part of the analysis workflow [7]. Without pre-processing, spreadsheet and business intelligence (BI) tools do not provide efficient aggregation or visualization. HR specialists wish to extract several attributes from the applicants’ data to focus on further trend analysis rather than to spend much time normalizing the data. A visualization system is the most effective way to address this issue without writing any code.

With the discussion with several HR specialists, their requirements for the visualization system are as follows: (A) A user should be able to choose the attributes to be used for further analysis. (B) A user does not want to miss out a smaller number of attributes, i.e., rare cases that are often excluded in quantitative analyses. (C) A user can compare attributes across multiple years. (D) A user can explore an overview of the data based on user’s criteria. Without these four requirements, for example, finding multi-year trends on prospective employees applying for a position in the HR department becomes difficult, since they represent a minute proportion of the entire prospective employees. To the best of our knowledge, there is no existing tool that satisfies these HR data analysis requirements.

**Fig 1:**
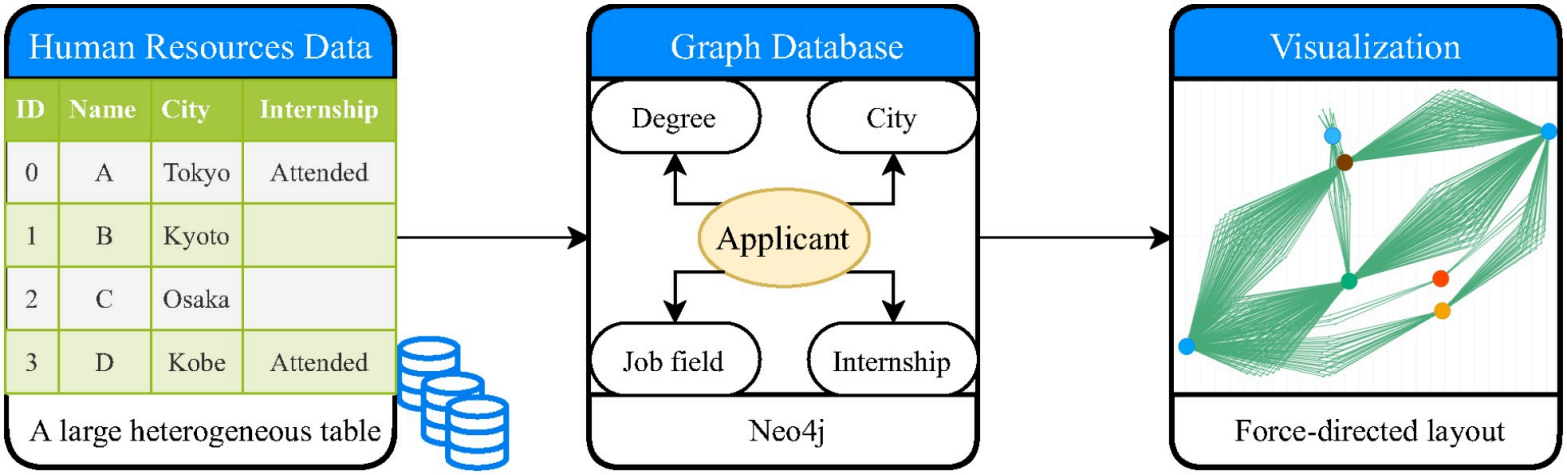
Data processing pipeline of the system. The input is a large data table of a recruitment database. We convert the input data as a single property graph stored in a graph database. The graph describes the relationship between prospective employees and their attributes such as skill, age, and university. The proposed method is a web application that visualizes the relationship between two attributes of applicants from the graph database.

Here, we propose the Polarizing Attributes for Network Analysis of Correlation on Entities Association (Panacea) visualization system. The system provides an interactive interface to explore every combination of two attributes of prospective employees each year. We employ property graph (PG) [8] as the data structure to provide more intuitive visualization for heterogeneous data. The proposed system satisfies the requirements (A)-(D), which are described in the design requirement section. The workflow for running the system is as follows (Fig 1): (1) convert the tabular data to a time-varying graph data; (2) extract subgraphs by user-specified attributes and years; and (3) draw the subgraph on an interactive interface.

To achieve this, a combination of visualization of dynamic time-varying graph and data-wrangling method to convert tabular data into a time-varying graph is needed. Although the conversion of tabular data into a graph has been explored previously [9–13], existing data wrangling methods do not explicitly encode timepoints on a graph. Moreover, dynamic graph visualization tools are not responsible for a graph conversion from temporal tabular data. We define a multi-partite graph to represent time-varying information of tabular data and then design an interactive interface to visualize dynamic graphs. Our primary contributions are:

- Time-varying graph modeling and a dynamic visualization system for heterogeneous tabular data with time scale.
- An interactive interface with dynamic graph visualization that satisfies the requirements of annual recruitment analysis.
- Three case studies and user studies demonstrating how HR specialists use the proposed system to analyze the database by finding unique trends.

## Related work

### Visualization for human resources

The HR data are stored in a tabular form in a relational database (RDB). To inspect such data, spreadsheet tools, such as Microsoft Excel and Google Sheets, would be the first choice. Those tools are often used to display the original data and perform fundamental aggregation, such as sorting, filtering, and visualizing using predefined charts. For example, Microsoft Excel is the most frequently used tool to manipulate tabular data for HR analysis [14]. Spreadsheet tools can compare two attributes at one point in time or they can show time-series variation in one type of attribute. However, such tools with the two-dimensional table representation are unsuitable for simultaneously analyzing two attributes with a time scale. Moreover, spreadsheet tools are not suitable for such heterogeneous or large tabular data due to aggregation and performance issues; thus, every time users must extract tables for their purposes.

Recently, BI tools such as Tableau, SpagoBI, and Qilk have been used for business analytics [15, 16]. For HR analysis, Tableau had been employed to analyze trends in job descriptions in New York using bar and line charts [17]. Although BI tools are useful for aggregation, there are two concerns. First, the backends of BI tools are based on the RDB model; thus, the data must be normalized, which requires a tremendous amount of effort. Second, once users specify the attributes to be analyzed, the tool can visualize them on a sophisticated interface. Users will employ BI tools effectively only if they can normalize all data and determine the types of visualization and aggregation that will be beneficial. Before starting tabular analysis and visualization, users need to choose the columns of focus.

If users have Python skills, they can use Jupyter Notebook [18] for inspecting data. Various kinds of visualization and inspection libraries are also available in Python. However, such libraries require advanced programming skill, thus hindering non-programmers from inspecting data by themselves.

Data heterogeneity is a major challenge for information visualization systems [19], including HR data analysis systems. Methods for visualizing HR data have been explored, and several visualization systems for HR data have been developed. Proactive [20] is a job description search engine with a rich interface. It displays a list of job descriptions in a tabular manner; however, the relevance between records is not shown on the interface. In addition, a system has been presented that displays each applicant with various metrics, such as talent or performance [21]. This system is useful for allowing users to know the attributes of each applicant; however, the relationships among attributes are not shown. In contrast, the proposed system focuses on the relationships among attributes of prospective employees.

### Visual analysis of categorical data

Many categorical, albeit unnormalized, data are stored in HR databases. State-of-the-art techniques of categorical data visualization have been reviewed in previous research [22]. They categorized these techniques as Euler-based, overlays, node-link, matrix, aggregation, and scatter. The node-link diagram, also known as network graph, is used to depict categories and their elements as nodes and edges in a graph. The node-link diagram can visualize either the relationship between categories and elements as bipartite graph [23–25] or that between categories as Parallel Sets [26].

They described the advantage of the node-link diagram as highlighting elements as nodes and clustering nodes of each category, making it easy to understand. Radial Sets [25] locate the category nodes along the arc of the circle. Element nodes are located inside the circle, which belong to multiple categories. Parallel Sets [26] visualize categories as parallel coordinates plot and the frequencies of the combination of categories as edges between categories with an interactive interface. They aim to visualize complex data information in its entirety. However, first-time users might find the description difficult to understand. The major downside of a node-link diagram is that the crossing edges make it difficult to understand the diagram. Also, the number of edges in the graph is often limited to about hundreds because of increasing clutter [22]. Nevertheless, the node-link diagram can be integrated with dynamic graph visualization for temporal data. We utilize the node-link diagram for visualization of categorical heterogeneous tabular data.

### Visualization for dynamic graphs

Several static graph visualization tools for general purpose, including Cytoscape, GraphViz, and Gephi [27–29], provide sophisticated methods to visualize any type of graphs; however, these tools were not designed to render dynamic graphs. Review articles have described a hierarchical taxonomy for categorizing visualization techniques for dynamic graphs [30, 31]. It can be subdivided into four types: (1) timeline node-link, (2) timeline matrix, (3) animation with a special-purpose layout, and (4) animation with a general-purpose layout. Hereafter, we describe each layout. (1) Timeline node-link approaches, such as the work of Greilich et al. (2009) [32] and Burch et al. (2011) [33], show superimposed or juxtaposed nodes for each year, which visualizes the relationship between nodes rather than the graph topology. Small multiples are categorized as the timeline node-link approach. (2) Timeline matrix approaches, such as the work of Stein et al. (2010) [34] and Burch et al. (2013) [35], are suitable for dense graphs; however, our target graphs are not dense as can be seen by the construction method. Timeline approaches visualize the time scale on the view, which requires a summarization of each time step. (3) Animation with a special-purpose layout can visualize graphs of each time step; however, the approaches need an abstract representation of nodes based on hierarchy or clusters. (4) Animation with a general-purpose layout can visualize graphs by general methods, and users can easily trace the position of nodes in each time step [36, 37]. We utilize (4) the animation general-purpose layout in the proposed system because it provides the most flexible visualization without any abstraction for each time step.

For selecting the method for visualizing dynamic graphs, the ability of the method to preserve the user’s mental image of the graph, i.e., mental map, is an important criterion [30]. The animation approach is one of the effective methods that provide a mental map by maintaining coherency among time steps [38, 39]. The animation approach enables users to gain more insight from changes between subsequent years in several cases, as demonstrated by Boyandin et al. (2012) [40]. However, as described by Hajij et al. (2018) [41], the limitation is that the graph topology can be lost on each timepoint. Several approaches to mitigate this limitation are available. For example, GraphDiaries [42] displays an animated transition of graphs between time steps, where disappearing nodes are highlighted first, followed by appearing nodes. Similarly, TempoVis [43] displays the color difference between appearing and disappearing nodes. Using a force-directed approach also mitigates the overhead of transition identification through time steps [44]. This is because this approach is able to trace the moving positions of the nodes of the previous timepoint. We utilize the force-directed approach for tracking the position of nodes.

### Graph visualization for tabular data

The efficiency of graph-based visualization for tabular data has been discussed regarding Orion [9], Ploceus [10], Graphiti [11], Origraph [12], and Oniongraph [13]. These are data-wrangling tools to convert tabular data into graph and can provide a graph visualization interface. These tools based on the concept that the network structure in tabular data can be represented as graphs, and they support constructing a graph from tabular data interactively. Orion uses domain-specific languages and a visual interface to display a node-link diagram without any attributes. Ploceus and the following tools explicitly support attaching attributes on each node. Further, several tools support a multi-layer graph. For example, Graphiti handles a multi-layer graph whose layers have different types of edges. OnionGraph uses node aggregation for hierarchical abstraction and provides a filtering function for nodes or edges. Both tools assume that the topology among layers is maintained. However, the time-varying graph does not assure that the graph topology is maintained through multiple timepoints. Therefore, special care to maintain a mental map is needed.

The temporal graph visualization has been adopted for some special cases. For example, MatrixFlow [45] is used for medical data and visualizes a temporal network as timeline matrix approach. ecoxight [46] visualizes business ecosystems as an animated timeline node-link diagram, though the input must be a graph.

Although Panacea shares the same concept in handling tabular data as a graph, we focus on the temporal data visualization for temporal tabular data. For this study, we wrote custom scripts to convert the graph data and developed a visualization system to explicitly display temporal information on the node-link diagram.

### Graph database

The selection of a graph database is a design decision. Categorical data visualization by a node-link diagram internally converts categorical data into a graph-based representation. Storing all categories into a graph database is a persistent and scalable way to query from the frontend each time. To store graph data in a graph-based database, mainly two definitions of graphs are available, i.e., the resource description framework (RDF) [47] and property graph (PG) [8]. RDF is a normalized data structure to describe relations between entities. PG is a more flexible graph format and is compatible with several graph databases, including Neo4j (https://neo4j.com). RDF is well normalized but is occasionally complex to visualize and to query because all entities must be nodes. Therefore, in the proposed system, we utilize PG as the data format for storing graphs.

Further, we employed Neo4j as a backend graph database. Neo4j has a company-provided visualization tool, i.e., the Neo4j browser. However, we needed to hold the position of specified nodes for preserving the mental map. Since the Neo4j browser does not support this feature, we wrote a custom frontend with vis.js (https://visjs.org). Several studies have implemented their own visualization system on top of Neo4j. For example, Onoue et al. (2018) [48] employed Neo4j as a backend graph database, and Caldarola and Rinaldi (2016) [49] used Cytoscape but stored data using Neo4j. Neo4j is state-of-the-art software to employ as the backend of the visualization system. We thus implemented our custom visualization modules on top of Neo4j.

## Design requirements

The primary goal of annual recruitment data analytics is to find trends or biases in historical data of prospective employees over multiple years. We invited three HR specialists from Panasonic Corporation to biweekly discussions (1-1.5 hours per meeting) on user requirement collection and prototype evaluations. We updated the system iteratively, which facilitated quick access to their feedback. Since different HR specialists have different goals for analyzing data, no typical analysis workflow is indicated. From the series of interviews with HR specialists, we extracted common procedures and summarized them as follows: (1) extracting subtables by specified attributes, years, and/or applicants; (2) data analysis and visualization of the extracted subtables; and (3) discussion on the results. Among them, (1) is a major obstacle for HR specialists because the heterogeneity of table columns requires much pre-processing effort and makes it difficult to provide an overview. HR specialists demand a system that helps them extract subtables without writing any code.

Further, we identified four challenges of extracting subtables. (A) It is difficult to select the columns for focus when extracting subtables. The combination of two columns is much larger than the number of the column; thus, it is difficult even for HR specialists to know which columns to use in subsequent analysis. HR specialists require an interactive system to examine the entire table prior to extracting subtables. (B) Pre-processing might cause abstraction or summarization of data, thus obscuring the relationship between the original data and the visualization. Such methods often ignore the smaller number of attributes. However, these ignored attributes, i.e., rare cases or outliers, are sometimes important for HR analysis. For example, the number of applicants who can speak multiple languages is not high; however, such skills are valuable when a company seeks to expand its business to global markets. This prevents further exploration into the original data when HR specialists wish to gain more insight. HR specialists require a visualization system that preserves the original data to avoid omitting the number attributes. (C) Applicants differ between years; however, most attributes are similar. HR specialists wish to analyze changes in the distribution of attributes over the years to identify trends or biases. (D) Different HR specialists have different criteria to perform analysis; thus, a criterion that regulates the visualization must be customizable. For example, the attributes clustering pattern depends on the choice of each HR expert. As a summary of (A)-(D), HR specialists require an integrated system with (A) a bird’s eye view that (B) does not omit outliers and (C) a time-varying view permitting (D) user customization.

Herein, we discuss the visualization method employed in the proposed system. For (A) a bird’s eye view (B) without omitting outliers, the advantage of a node-link diagram, which can highlight elements and is easy to understand, is indispensable. Using the node-link diagram, nodes can be visualized without aggregation. Spreadsheet or BI tools partly support these requirements but require pre-processing. Matrix, scatter plot, and flow-based visualizations provide a one-versus-one comparison, e.g., one attribute versus another attribute, or one attribute on the time axis. In such cases, the target attributes must be mapped to a single axis to align them horizontally or vertically, thereby incurring actual data loss in the relationship between attributes. These data should be handled with minimal modification or aggregation from the data stored in the database. Therefore, we employ PG as a data structure and a node-link diagram for categorical data visualization. For providing (C) a time-varying view, there are several options to integrate into the graph visualization, e.g., timeline (small multiples) or animation. Among them, the animation approach enables users to trace the transition between even distant years, thus keeping their mental map and providing them with more findings [38, 40]. At last, enabling users to move the positions of the nodes corresponds to (D) user customization. The entire system is described in the next section.

## Implementation

**Fig 2:**
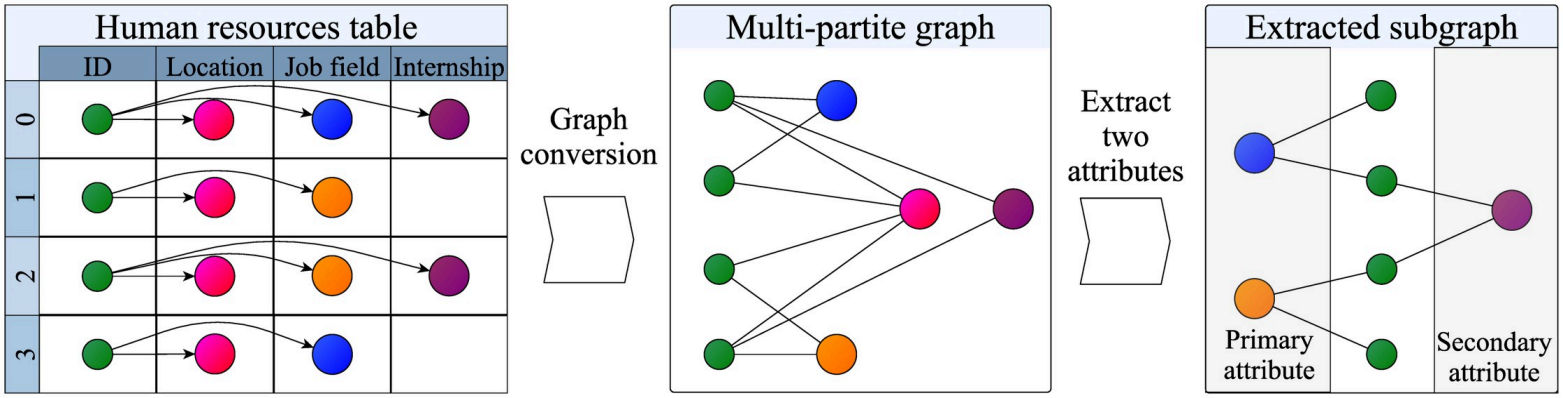
Data model in Panacea. First, the pre-processing script converts all tabular data into a single multi-partite graph. Next, users select two attributes and then query the backend server. The backend server returns a subgraph, including records of applicants and two corresponding attributes from the backend server.

The proposed Panacea system is a web application with three components (Fig 1): data pre-processing, a backend server, and an interactive frontend. For data pre-processing, we propose a data model based on a multi-partite graph and write custom scripts to convert a table to graph representation (Fig 2). Here, we use PG exchange format as an intermediate output of data processing [50]. Further, we employ Neo4j as a backend server and X2 (https://github.com/g2glab/x2) as middleware for visualization. JavaScript and vis.js are used as the visualization library on the frontend.

### Data model and preprocessing

Multi-partite graph G is expressed as *G* = ((*A*, *V*_1_, *V*_2_, …, *V*_*n*_), *E*), where *E* ⊆ *A* × *V*_*i*_ such that *A* represents applicants, each of *V*_1_, *V*_2_, …, *V*_*n*_ is an applicant attribute, and *E* represents the relationships between an attribute and applicant. Each record in the table corresponds to a single applicant node. There is an edge *e* ∈ *E* if and only if the applicant *a* ∈ *A* has an attribute *v* ∈ *V*_*n*_. From the frontend, users select two attributes *V*_*x*_, *V*_*y*_ from *V*_1_, *V*_2_, …, *V*_*n*_. Queries to retrieve subgraphs are encoded in Cypher, which is a query language for Neo4j. Based on the definition of PG, nodes can have properties. Here we set the *type* property of each attribute node to describe the category name of attributes, e.g., *academic credential* or *internship history*. We also set the *year* property of each applicant node to specify the year to explicitly encode the time scale on a graph.

Since we have already determined the design requirements, defining a custom conversion to satisfy these requirements is more reasonable than writing domain-specific languages or using data wrangling tools. Indeed, manual curation using existing data wrangling tools is not practically suitable to convert large and temporal tabular data into a time-varying graph. Therefore, we implement novel custom scripts to encode the temporal tabular data into a time-varying multi-partite graph.

In the HR database, each row is an applicant, and each column is an attribute. We simply regard each column as (1) a single-column attribute, (2) a property of the applicant node, or (3) a multi-column attribute. For example, *academic credential* should be assigned as (1). *Name* should be categorized as (2) because *name* is tightly linked to the attribute; therefore we do not want to regard *name* as an independent attribute. Let us consider *internship history* as an example of (3). There are three columns labeled as *internship history1*, *internship history2*, and *internship history3*. These columns contain company names as string datatype where applicants worked as an intern. These columns should not be assigned as independent attributes because the three columns are just an inflated array of *internship histories*. Thus, we assign these columns to the same type on attribute nodes to merge these columns into one category. Such columns represent poor database design because the columns are not normalized. Instead, we should have an internship company table with a unique key for each company and an intermediate table with two keys for applicants and companies. However, we could not modify the original table structure due to the limitations of the recruitment management system. Most of the columns are categorized as (1), but we find that several columns should be categorized as (2) or (3). There are several models to convert tabular data to PG [51, 52]; however, they do not convert (3) a multi-column attribute. The advantage of graph representation is that graph can handle such kinds of unnormalized relational data smoothly.

The entire procedure to convert the HR data into the graph is as follows. An empty graph *G* is initialized, and the following procedure is repeated for each record in the table: First, the applicant node *a* ∈ *A* is inserted into *G* with properties from all elements that are a property (2). Next, for all elements that are an attribute node (1) or (3), a tuple of two nodes and an edge (*a*, *e*, *v*) *s*.*t*. *a* ∈ *A*, *e* ∈ *E*, *v* ∈ *V*_1_, …, *V*_*n*_ is inserted into *G*. At last, graph *G* is imported into Neo4j.

### Overview of systems

**Fig 3:**
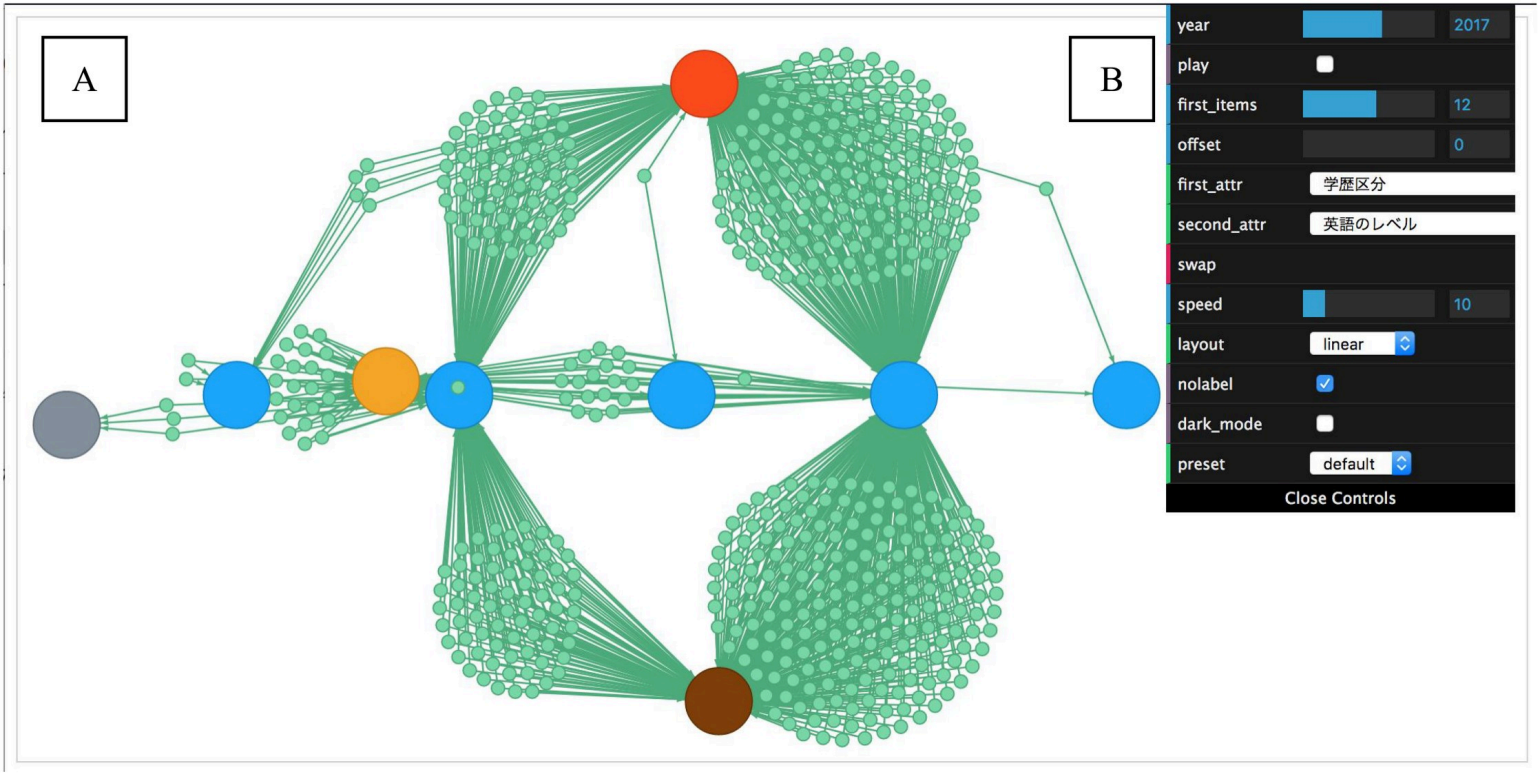
Screenshot of Panacea. The interface has (A) graph and (B) configuration views. The graph view shows an example of the relationship between two attributes. The small green nodes are applicants; the large blue nodes are primary attributes; and the other large nodes are secondary attributes. The green edges represent relationships between applicants and attributes.

Panacea’s frontend has two view modules, i.e., graph and configuration views, as shown in Fig 3. The graph view visualizes two attributes and temporal information between two arbitrary timepoints. With the combination of components, we provide an “overview first, zoom and filter, then details-on-demand” system, as introduced in Shneiderman’s Visual Information-Seeking Mantra [53]. We can visualize two attributes throughout the timepoint. Two attributes are classified as primary and secondary attributes. The primary attribute is highlighted by using a color or position on the graph view, thus working as a criterion to be compared with the secondary attribute.

The subgraph *G*(*year*, *x*, *y*) = ((*A*_*year*_, *V*_*x*_, *V*_*y*_), *E*) is displayed in the graph view (Fig 3A). Here, each attribute node has a Japanese label (omitted in all figures; English labels were superimposed as necessary). Each applicant node is not labeled because the applicant nodes vary across years. The primary attributes *V*_*x*_ are displayed with *star* layout, which places the node with the maximum degree at the center, and the remaining nodes surround the central node on the circumference. Then, *A* and *V*_*y*_ are visualized using a force-directed layout with the ForceAtlas2 algorithm [54]. *V*_*x*_ and *V*_*y*_ are large nodes with different appearances. *A* is visualized as small green nodes. Users wish to see what attributes that an applicant has, especially if the applicant appears as an outlier due to the node location. A small pop-up appears to show the entire list of an applicant’s attributes when users click *A*. Edge *E* connects between *A* and *V*. Due to the limitations of performance and perception, we do not recommend visualizing more than a hundred nodes at the same time. *Limit of primary attributes* and *offset of primary attributes* parameters are useful for reducing the number of entire nodes. Since primary attributes are sorted by the occurrence of each attribute, users can retrieve subgraphs including an arbitrary range of primary attributes.

The force-directed layout calculates the positions of nodes based on physical simulations. Gravity makes two nodes closer, whereas repulsion makes two nodes more distant. All nodes are separated due to repulsion between nodes, but node pairs connected by an edge make closer. As more and more edges connect nodes, the connected nodes can be closer. As a result, the distance between attribute nodes is adjusted across the entire graph, which helps users to see the relationship between attributes.

The primary attributes are anchored on the initial position, whereas the secondary attributes and applicant nodes can move. Only users can move primary attribute nodes to an arbitrary position, which enables them to perform manual clustering of nodes based on a category, feature, etc. When users move the position of a primary attribute node, the position of the remaining nodes is recalculated and moves at the same time. Our initial implementation was to visualize all nodes using the force-directed layout without constraints on positions. However, the visualization between the primary or secondary attributes was obscure because the two attributes were superimposed or mixed. To avoid this, we suspend the position of the primary attribute. Further, the graph can be zoomed in/out using a mouse scroll. With the force-directed layout and zooming, users can intuitively see correlations among the primary and secondary attributes.

The animated force-directed layout enables users to observe the transition across multiple years. When users select a year to be displayed, applicant nodes and all edges are removed and re-rendered. We employ two techniques to preserve the mental map by reducing differences between timepoints. First, the positions of the primary and secondary attribute nodes are maintained at this time. Second, the applicant nodes are maintained if and only if the target node has the same edges over the years. Then, all edges are drawn between nodes, and the positions of secondary attributes and applicant nodes begin to change. This animation illustrates the dynamic transition across different years, which helps users to see the trends. The transition is not limited to consecutive years, i.e., the users can observe dynamic differences between distant years.

#### Configuration view

The configuration view (Fig 3B) allows users to select two types of attributes, *V*_*x*_ and *V*_*y*_, from a dropdown menu. We assume that the users are HR specialists; thus, they select attributes based on their experience and insight. Here, the users select primary attributes *V*_*x*_ and secondary attributes *V*_*y*_. Afterward, *G*(*year*, *x*, *y*) = ((*A*_*year*_, *V*_*x*_, *V*_*y*_), *E*) is displayed in the graph view. The users can also specify other parameters to change the appearance of the graph view. We enumerate some of the parameters as follows:

- *year*: Users must specify the fiscal year (FY) to visualize using a slider or input text.
- *limit of primary attributes*: Users can limit the number of primary attributes nodes to display.
- *offset of primary attributes*: Users can fetch the primary attributes nodes, skipping the specified number of attributes.
- *auto play*: When users enable autoplay mode, the dynamic transition of the graph is automatically played.

## Case studies

We now apply the proposed system to a real-world dataset, an HR applicant data. Applicant data in the FY 2014-2020 were provided by Panasonic Corporation. The applicant data were dumped in isolated comma-separated values files for each fiscal year. The total number of users who registered with the system was about 400, 000; however, most registered to the company’s system but did not apply for a position in the company. The total number of applicants that obtained employment was about 4, 000, which is the focus of this study. The number of columns ranged from about 3, 000 to 7, 000 depending on the year, and only 1, 100 columns were shared across the seven-year period. This means that other columns were not the same across the same period due to changes in a database schema. Therefore, we wrote custom scripts for matching columns with names that were changed across the years.

Although the proposed system can support all enumerable values in a column, we selected 12 columns during user evaluation based on suggestions from HR specialists. HR specialists focused on the following six columns (applicant’s attributes) in the case studies.

- *Applicant ID*: Universal unique ID for all applicants.
- *Location of university*: Nine regions (one overseas region and eight regions in Japan).
- *Academic credential*: Bachelor, master, or doctoral student. We also categorized new or previous graduates.
- *Self-declared English skill*: Applicants select from [Entry, Conversational, Business, Native]-level English skills.
- *Job field*: Jobs at Panasonic Corporation (sales, system engineer, research, HR, etc.) for which applicants applied.
- *Internship history*: Names of companies where applicants worked as interns.

All analyses were performed in Panasonic Corporation and the data were provided by Panasonic Corporation. Thus, the research was not checked by an institutional Ethics Committee, while this research was performed under the permission of Panasonic Corporation. Informed consent has not been conducted since all data were analyzed anonymously. Potentially identifying information (name, address, phone number, e-mail address, etc.) were removed or masked. All the testers invited in expert feedback were informed that the case studies were to be published.

### Example A. Location of university versus self-declared english skill

**Fig 4:**
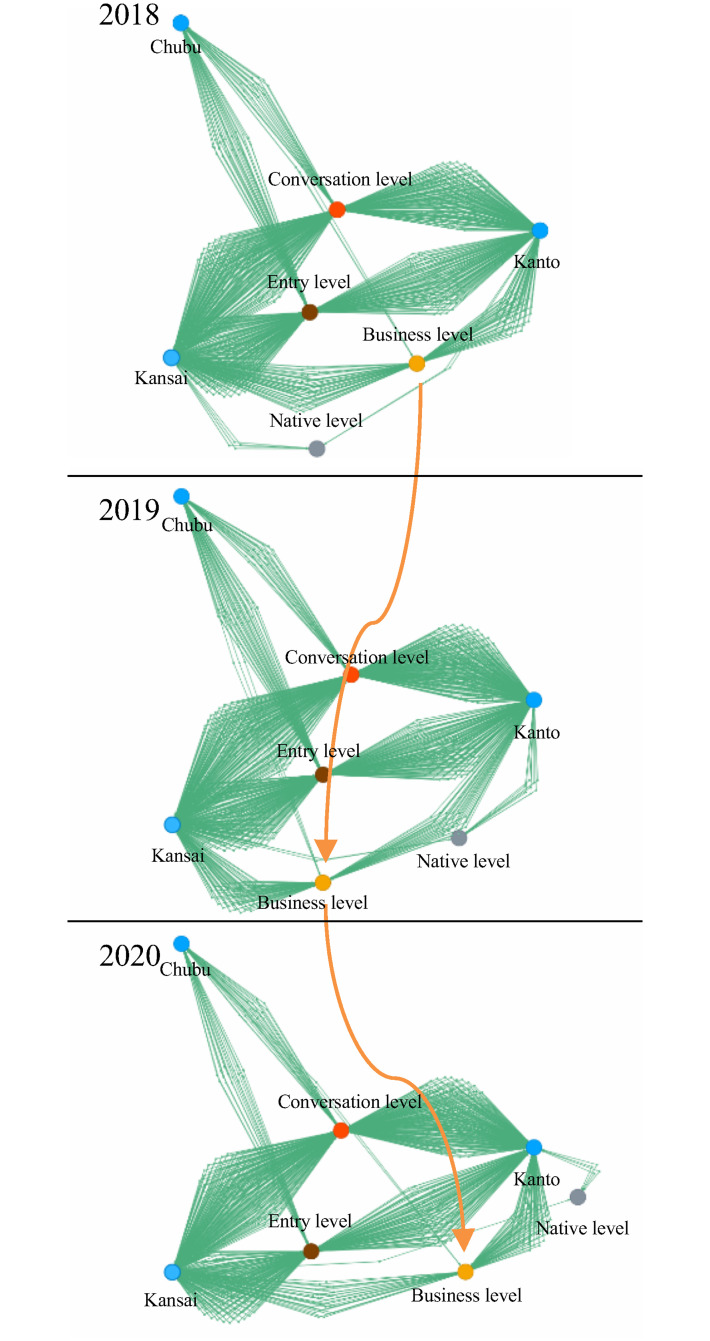
Example showing *location of university* versus *self-declared English skill* from FY 2018 to 2020.

We present the first case study to demonstrate the transition over three years using the animated force-layout rendering in Panacea. Fig 4 shows the graph view between *location of university* versus *self-declared English skill* of prospective employees. Here, the three large blue nodes correspond to three regions in Japan, i.e., Kanto, Kansai, and Chubu, which are arranged in a circle. Kanto is the central-eastern part of Japan’s main island and includes Tokyo. Kansai is the central-western area and includes Osaka. Chubu is the central area and includes Nagoya. The large colored nodes (except for the blue nodes) are self-declared English skills collected from the submitted resumes, and the small green nodes are prospective employees. In this case, the colors differ based on the applicant’s level of English: brown is entry-level, orange is conversational, yellow is business, and gray is native. In FY 2018 and 2020, the yellow node (business level English skills) is generally located between two blue nodes. However, the yellow node moved from the blue node Kanto to the blue node Kansai in FY 2019. This shows a trend in FY 2019 such that there were more employees with business-level English skills in Kansai, but fewer applicants had the same skill in Kanto and Chubu. Further, the gray node moves dynamically because its degree is less than that of the other large nodes. We also observe an attribute with a small number of applicants, which is often ignored by quantitative analyses. This result is an example of trend observation via time-varying graph visualization.

### Example B. Major in university (liberal arts or sciences) versus job field

**Fig 5:**
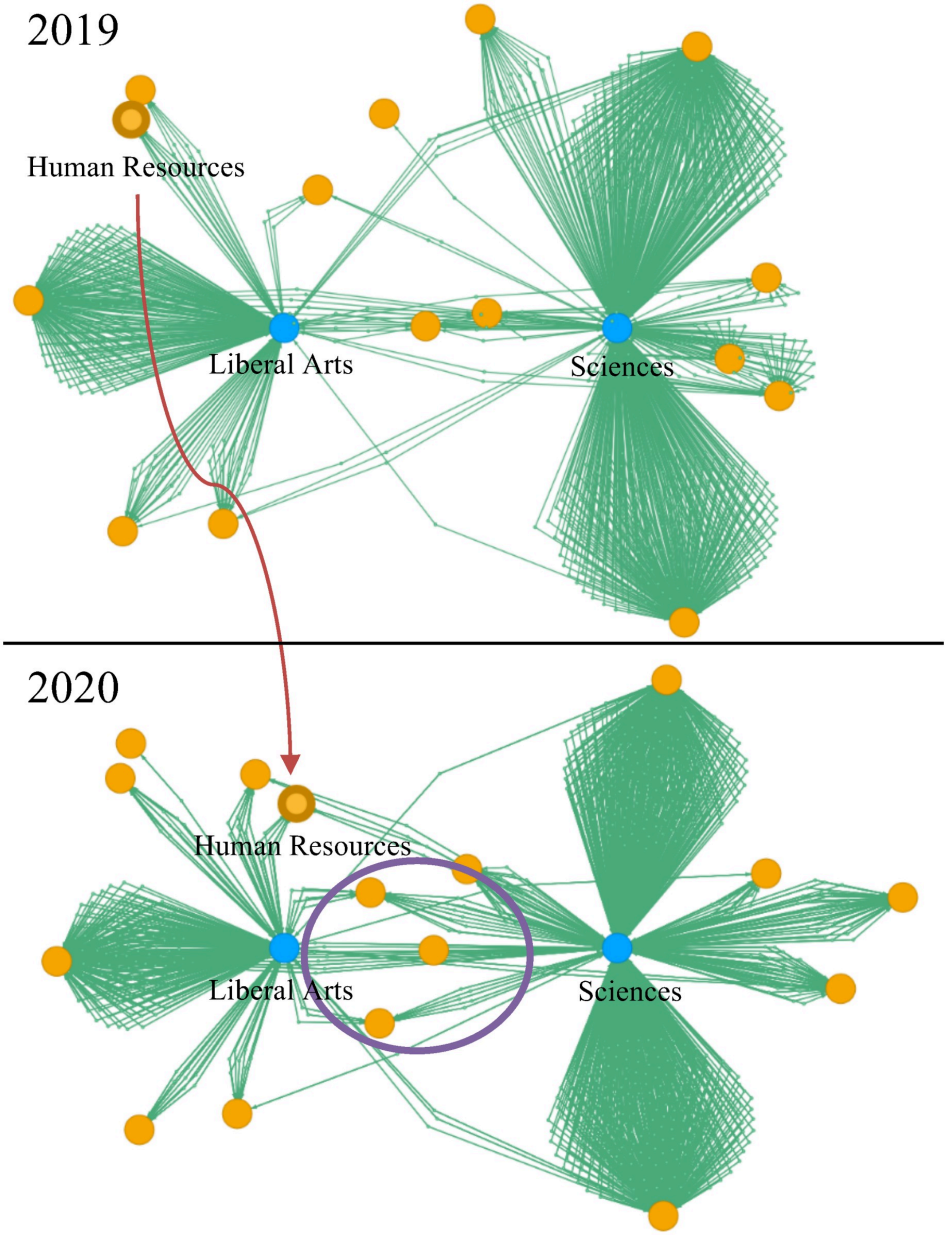
Example showing the transition of *major in university* versus *job field* from FY 2019 to 2020.

Fig 5 shows an example of integration with prior knowledge of HR specialists and the corresponding visualization. Traditionally, the selection process in most companies in Japan depends on the student’s future occupational class. Students majoring in social sciences, law, or humanities (referred to as liberal arts) will enter administrative jobs such as accounting, HR, personnel, sales, or purchasing. Students majoring in engineering or sciences (referred to as sciences) will enter technical jobs [4]. They are assigned a department after job training in a company for several months. Panasonic Corporation had employed applicants in administrative or technical jobs, mainly. Recently, Panasonic Corporation has started another recruitment policy where each department directly hires applicants. This example shows the transition of *major in university* versus *job field* from FY 2019 to 2020.

Here, the two blue nodes are *major in university* (liberal arts or sciences). The highlighted orange node is the HR department. The blue node connected to the highlighted orange node in FY 2019 is the liberal arts node. This indicates that all new employees who were assigned to the HR department in FY 2019 majored in liberal arts. However, in FY 2020, the highlighted orange node moves to the middle of the two blue nodes (shown by the red arrow), which indicates that the HR department began to employ people who majored in sciences or engineering. Similarly, three nodes are shown between the two blue nodes (shown in the purple circle). These three job fields employed people who majored in either liberal arts or sciences. The number of orange nodes located between the two blue nodes indicates that an increasing number of job fields tends to employ applicants regardless of their university major. Note that this trend does not mean that university majors are not considered; rather, it indicates that diverse expertise is in demand in various job fields. These results show an example of observing the changes in recruitment policies for job fields.

### Example C. Academic credential versus self-declared english skill

**Fig 6:**
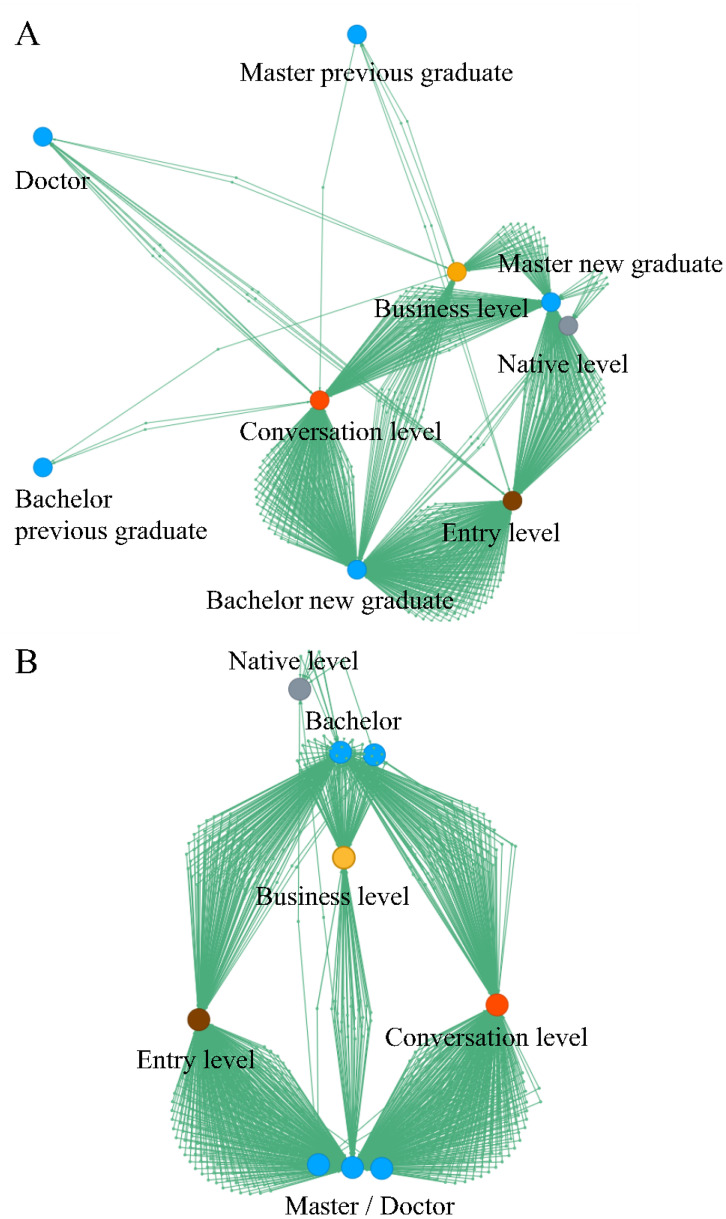
Example showing *academic credential* versus *self-declared English skill* of prospective employees: (A) initial layout; (B) layout after manual clustering.

Fig 6 shows an example of the manual clustering of primary attributes. Here, the primary attribute is *academic credential*, and the secondary attribute is *self-declared English skills*. In the original data, the *academic credential* fields store five values: bachelor’s degree new graduate, bachelor’s previous graduate, master’s new graduate, master’s previous graduate, and doctoral graduate. This is because Japanese recruitment custom distinguishes applicants by degrees and graduation time. Fig 6A shows the initial layout with five primary attribute nodes (shown in blue) and four secondary attribute nodes (shown in red, orange, gray, and brown). Due to differences in the number of applicants in each primary attribute, relationships become difficult to interpret, which is a non-negligible burden for visual understanding.

Panacea allows the users to easily move the positions of the primary nodes. HR specialists move the position of the primary nodes to two distant locations in Fig 6B. This procedure is a kind of clustering because the two distant positions can be seen as two clusters, i.e., bachelor’s and master’s/doctoral, which emphasizes the difference between degrees by ignoring the difference between new or previous graduates. Here, the top cluster is bachelor’s, and the bottom cluster is master’s/doctoral. The gray node is a native speaker, and the yellow node is a business-level English speaker. As can be seen, most native and business-level speakers have the academic credentials of bachelor. This trend is not unexpected because most master’s/doctoral students are employed in technical jobs and most work in factories or research centers in Japan. In contrast, a large part of bachelor’s students are employed in administrative jobs, including international sales, purchasing, and HR. English skills are one of the criteria for assigning prospective employees to these positions. This example with the manual clustering function is more attractive for HR specialists. Users can also cluster graphs based on other criteria, e.g., whether applicants have already graduated. The proposed system can combine such empirical knowledge with data visualization.

### Expert feedback

We invited three HR specialists (denoted T1, T2, and T3) from various positions in an HR department as testers to evaluate the proposed system. To mitigate bias, these HR specialists were not the same as those who participated in the previously discussed biweekly meetings. The user evaluation phase lasted an hour per person. We made a presentation (this lasted for 20 minutes) explaining how to use the proposed system, and then we watched how the users tested this system and interviewed the user (this lasted for 40 minutes). We observed that the users operated the system without special training. The questions we asked the users during the interview are as follows.

(Q1) How did you feel when you used the system? (General usability)
(Q2) Which part of the system was helpful for information discovery? (Visual design)
(Q3) Which part of the system did you find unfamiliar to use at first instance? (System usability)
(Q4) What additional features do you think could be integrated into the system to help with further knowledge discovery? (Additional data source)
(Q5) What did you discover using this system? (Knowledge discovery)

We summarized the testers’ responses as follows:

(A1) They had a good impression of the system because this kind of visualization was novel to them, and they could intuitively understand the relationship between attributes.
(A2) The graph view is the most important component because the topology and transition of the graph are displayed simultaneously, which helps them recognize trends.
(A3) What surprised the testers the most was that the primary nodes can move everywhere, although several testers did not recognize this function at first. With this function, the testers clustered primary attributes based on their criteria. For example, a university can be categorized by grade or location depending on what the tester wishes to observe. Thus, clustering nodes based on individual criteria attracted more interest than the provided layout.
(A4) After the user evaluation, the testers requested to import other data. For example, they would like to visualize the time-varying visualization on the snapshot of each day, which means the transition of the daily trend during a single recruitment period. The daily log must be stored in the database to track daily changes. However, such data are currently not stored in the database because it stores only finalized information. As a result, the testers realized that they must have a log tracking system during the recruitment period.
(A5) They all achieved knowledge discovery through the system, as described in several case studies in this section.

The testers noticed two issues with the attribute normalization. (1) Several job fields that shared similar names disappeared and appeared at the same time during the transition between two years. However, some of these appear to be artifacts. Indeed, the restructuring of the organization has occasionally led to changes in the names of several divisions. (2) Unnormalized data were stored in the database. Even in a snapshot of a specified year, the users observed several different words representing the same object in the internship history attribute. This issue is often referred to as “employer normalization” [55, 56], which requires effort to annotate the different name entities to be the same. This unexpected appearance of the graph also suggests room for improvement to highlight the disappearing or appearing nodes, as seen in TempoVis or GraphDiaries [42, 43]. Through the proposed system, unnormalized data could be detected via unexpected appearance in the graph layout.

We summarized the comments from each tester as follows. T1, who is a section manager in the recruitment branding team, appreciated the overview of two specified attributes, which mitigated their burden compared with the conventional use of spreadsheets or BI tools. T1 suggested that the system would be useful for continuous improvement of employment policies because the system could visualize trends from all available data. T2, who is a chief of the recruitment team for technical jobs, highlighted that the number of the edges corresponds to the correlation between two attributes, which provides clues on how to inspect these two attributes extensively. T3, who was assigned to the HR department only three months prior to the test, easily caught up with the latest recruitment trends. T3 highlighted that the dynamic visualization of time-varying graphs shows the volatility of each attribute, which contributes to recognizing changes in trends. These comments indicate that the system was widely accepted by the testers regardless of their expertise or position.

## Discussion

The Panacea system proposes a time-varying multi-partite graph model for converting tabular data to the graph to apply dynamic graph visualization methods. Existing data-wrangling methods were not sufficient to generalize a graph model and visualization to temporal heterogeneous tabular data. Therefore, we modeled the multi-partite time-varying graph model and adapted the dynamic graph visualization to that graph. This is a remarkable user study for the integrated system of the time-varying graph model converted from heterogeneous HR data and visualization designed for that model. The results also underscore the pressing need for a general data wrangling framework of the time-varying graph model and dynamic visualization for temporal tabular data.

One of the main goals of the Panacea system is to provide HR specialists with interactive visualization so that they can discover the time-varying relationship on applicants’ history. The multi-partite graph visualization enabled HR specialists to focus on both the temporal changes of trends and the attributes of each applicant.

The combination of visualization techniques is novel for HR data analysis and enables HR specialists to gain new insight into this process. Most importantly, HR specialists noticed that several columns are stored in undesirable ways. Such observations would contribute to improving the recruitment management system for next year’s recruitment process. This system is a design guide for future development of an HR data analysis system.

The categorical characteristics of the HR database and the schema-less graph database underscore the importance to handle HR data as a graph. In general, relational databases require a schema, whereas graph databases do not require one in advance. Thus, there is no need to migrate the graph database schema even if the table schema will be changed next year. The system can visualize new data as soon as the new data is imported into the system. We also expect that the system can be integrated with other relational databases.

The force-directed layout calculates node positions based on a spring model through the entire graph; therefore, nodes move randomly and sometimes do not converge. This is something of a disadvantage of the layout. To mitigate this, we hold the positions of several nodes across multiple years. Also, we reuse node positions between each timepoint to suppress random movement. Although the layout requires careful interpretation of the results, the simple dynamic animation makes it easier for HR specialists to grasp the overview of trends each year.

We added several constraints to the visualization to make the system more interactive and intuitive, and these constraints were needed. The maximum number of applicant nodes shown at the same time would not be exceeded a few hundred. Since applicants vary each year, applicants from different years are not displayed at the same time. Therefore, the system can visualize at most a few thousands of applicants in total duration. However, the prospective employees of new graduates are a few thousand in total on Panasonic Corporation. For the initial analysis, the system is found to be sufficient. Further, we limited the number of attributes that can be observed simultaneously to two. Several relationships can exist between three or more attributes; however, these relationships are reduced to a one-to-one relationship between two attributes. This enables the users to find the trends between attributes.

### Limitations and future works

We demonstrated Panacea’s usability in a large Japanese company. The system can also be extended to other countries and other large companies with a closer number of prospective employees. However, there is still room for improvement for all applicants to be inspected. Note that applicants are selected and filtered out as the recruitment process proceeds. As a result, the database becomes sparse, which is more suitable for the graph structure than tables. Meanwhile, we need to explore the visualization technique for a larger number of applicants. Furthermore, we need to explore better shape and color of attribute nodes if the number of attributes increases. Nevertheless, the scalability of the pre-processing and backend system is maintained even for future visualization updates owing to graph databases and modeling.

The Panacea system has the potential to employ other visualization techniques. Applicant nodes are useful for understanding the number of applicants, but this is sometimes redundant and raises performance issues. One method for mitigating this is to remove applicant nodes and to connect attribute nodes directly. Since the proposed system supports multi-column attributes, removing applicant nodes makes visualization difficult. If the degree of applicant nodes is restricted to two, we can easily remove the node and connect adjacent nodes using a single edge. However, removing a node whose degree is greater than three generates a hypergraph [57] whose edge connects greater than two nodes. In fact, if there is an inflated array such as *internship history* with greater than two items, the graph becomes a hypergraph by definition. Although a hypergraph is the generalization of the graph used here, rendering a hypergraph is more complicated than the current method. Thus, we do not employ a hypergraph approach. Another method is to bundle the same edges by merging applicant nodes whose edges have the same destination, like Parallel Sets [26] or RadialNets [25]. However, both methods lose a simple interface to see each applicant’s information when users click each node. There is room for exploring more effective visualization.

We showed that HR specialists can find trends from 12 columns. The data model and pre-processing pipeline can convert all of the columns into a multi-partite graph. However, an efficient way of selecting two attributes from more than thousands of columns has not been implemented, and the efficient way to provide this remains unknown. For example, categorizing names of columns requires the HR expert’s prior knowledge or natural language processing. At the moment, we display only 12 columns by depending on HR specialists’ prudent choice in advance. We tried to add a visualization module for selecting the attributes, but however, we found that it would not be effective without any categorization by HR specialists in advance. We plan to explore an effective recommendation method to help HR specialists select columns.

We succeeded in observing a few years of trends in the case studies. Since we aimed to visualize a long-term range transition, we applied the animation method to visualize the entire duration. Unfortunately, HR specialists were unable to observe any drastic transition in the trends throughout the entire duration using the system. We suspect that there was hardly any drastic transition of employment trends in Panasonic Corporation probably because the economic growth in Japan was stable and thus the unemployment rate was kept low [58]. Further, we did not evaluate the recruitment policies during the research period because the recruitment period in Japan is limited to once per year. These concepts are reserved for future work in the field of HR management.

One potential application of interactive graph clustering would be in tackling the problem of name identification such as employer normalization. Previous studies have employed heuristic and machine learning methods with manual annotation, web resources, and business database [55, 56]. Still, continuous manual effort is required. Name variants decrease the analysis reliability; however, curation for normalizing such entities is often time-consuming. Since unexpected data can be found more easily with the visualization, we suggest that further research should be undertaken in an interactive name identification system using graph visualization.

The proposed graph model can be extended to other future applications. For example, bibliometric networks are used for showing the relationship between articles [59], often visualized as a node-link diagram [60]. In most tools, bibliometric networks are represented as a homozygous graph (nodes are publication, edges are the relationship between publication). However, the categories of publications like keywords or author’s affiliation can be represented as primary/secondary nodes. Still, this would require a more general framework to convert data into graphs. The Panacea system is potentially extensible for other types of data sources by implementing data conversion and visualization modules based on the Panacea graph model.

## Conclusion

This study has proposed the Panacea system for HR specialists, which visualizes time-varying graphs from a heterogeneous table for the simultaneous recruitment of new graduates. To the best of our knowledge, this study is the first attempt to display large heterogeneous HR tabular data as a dynamic graph. Three case studies demonstrate the usability of time-varying graph animation with an interactive interface for inspecting heterogeneous databases. Future work should involve a general framework with a time-varying-graph-based data model and dynamic visualization for heterogeneous temporal tabular data.
